# Triple tSMS system (“SHIN jiba”) for non-invasive deep brain stimulation: a validation study in healthy subjects

**DOI:** 10.1186/s12984-022-01110-7

**Published:** 2022-11-24

**Authors:** Sumiya Shibata, Tatsunori Watanabe, Takuya Matsumoto, Keisuke Yunoki, Takayuki Horinouchi, Hikari Kirimoto, Jianxu Zhang, Hen Wang, Jinglong Wu, Hideaki Onishi, Tatsuya Mima

**Affiliations:** 1 Department of Physical Therapy, Niigata University of Health and Welfare, 1398 Shimami-Cho, Kita-Ku, Niigata-Shi, Niigata, 950-3198 Japan; 2 Institute for Human Movement and Medical Sciences, Niigata University of Health and Welfare, 1398 Shimami-Cho, Kita-Ku, Niigata-Shi, Niigata, 950-3198 Japan; 3 Faculty of Health Sciences, Aomori University of Health and Welfare, 58-1 Mase, Hamadate, Aomori, 030-8505 Japan; 4 Department of Sensorimotor Neuroscience, Graduate School of Biomedical and Health Sciences, Hiroshima University, 1-2-3 Kasumi, Minami-Ku, Hiroshima, 734-8553 Japan; 5 School of Mechatronical Engineering, Beijing Institute of Technology, No. 5 South Street, Zhongguancun, Haidian, Beijing, 100081 China; 6 Qingdao Medical College, Qingdao University, 308 Ning Xia Lu, Laoshan, Qingdao, 266071 Shandong China; 7 Graduate School of Interdisciplinary Science and Engineering in Health Systems, Okayama University, 1-1-1 Tsushimanaka, Kita-Ku, Okayama, 700-8530 Japan; 8 The Graduate School of Core Ethics and Frontier Sciences, Ritsumeikan University, 56-1, Tojiin, Kitamachi, Kita-Ku, Kyoto, 603-8577 Japan

**Keywords:** Transcranial static magnetic field stimulation, Non-invasive brain stimulation, Deep brain stimulation, Neodymium magnet, SHIN jiba

## Abstract

**Background:**

Transcranial static magnetic field stimulation (tSMS) using a small and strong neodymium (NdFeB) magnet can temporarily suppress brain functions below the magnet. It is a promising non-invasive brain stimulation modality because of its competitive advantages such as safety, simplicity, and low-cost. However, current tSMS is insufficient to effectively stimulate deep brain areas due to attenuation of the magnetic field with the distance from the magnet. The aim of this study was to develop a brand-new tSMS system for non-invasive deep brain stimulation.

**Methods:**

We designed and fabricated a triple tSMS system with three cylindrical NdFeB magnets placed close to each other. We compared the strength of magnetic field produced by the triple tSMS system with that by the current tSMS. Furthermore, to confirm its function, we stimulated the primary motor area in 17 healthy subjects with the triple tSMS for 20 min and assessed the cortical excitability using the motor evoked potential (MEP) obtained by transcranial magnetic stimulation.

**Results:**

Our triple tSMS system produced the magnetic field sufficient for neuromodulation up to 80 mm depth from the magnet surface, which was 30 mm deeper than the current tSMS system. In the stimulation experiment, the triple tSMS significantly reduced the MEP amplitude, demonstrating a successful inhibition of the M1 excitability in healthy subjects.

**Conclusion:**

Our triple tSMS system has an ability to produce an effective magnetic field in deep areas and to modulate the brain functions. It can be used for non-invasive deep brain stimulation.

## Background

Transcranial static magnetic field stimulation (tSMS) is a promising non-invasive brain stimulation modality because of its competitive advantages such as safety, simplicity, and low-cost [1, 2]. Using a small and strong neodymium (NdFeB) magnet, tSMS can inhibit cortical excitability just below the magnet [2–6] as well as modulate brain-wide network [7, 8] and brain functions remote from the magnet [9, 10]. Since the effects of tSMS are not directly associated with induced electric current, tSMS never provokes seizure or tingling skin sensations unlike transcranial magnetic stimulation (TMS) and transcranial electrical stimulation (tES). With these advantages, tSMS is recently used as self-administered daily treatment at home for a neurological disorder [11]. However, previous works on human brains with tSMS have been limited to stimulation over cortical surfaces [2–5]. Since the magnetic field strength decreases with the distance from the magnet, the current tSMS cannot produce a magnetic field sufficient to provoke biological effects in deep brain areas [12].

Deep brain areas such as the basal ganglia and hippocampus are involved in a number of movement, neurological, and psychiatric disorders. Some of these disorders can be treated with deep brain stimulation (DBS) delivering constant or intermittent electricity to a target located in the deep brain areas. Stimulation-induced disruption of pathological brain circuit activity has been proposed as a mechanism by which the DBS operates [13]. Although the DBS is a powerful tool for treatments of brain diseases, it has several disadvantages. First, it requires neurosurgical implantation of electrodes into deep brain structures. Such procedures are associated with serious surgical risks as well as a lifelong implant. Second, it incurs large financial costs for the special equipment. Therefore, it is important to develop a low-cost non-invasive deep brain stimulation system.

Here, we propose a triple tSMS system, colloquially termed as “SHIN jiba” in Japanese, enabling to stimulate deep brain areas with an effective magnetic field non-invasively. We demonstrated the triple tSMS could produce a magnetic field with a sufficient strength to provoke biological effects in a remote area from it. Further, we confirmed the triple tSMS could inhibit cortical excitability below it like the current single tSMS in healthy subjects.

## Methods

**Fig. 1:**
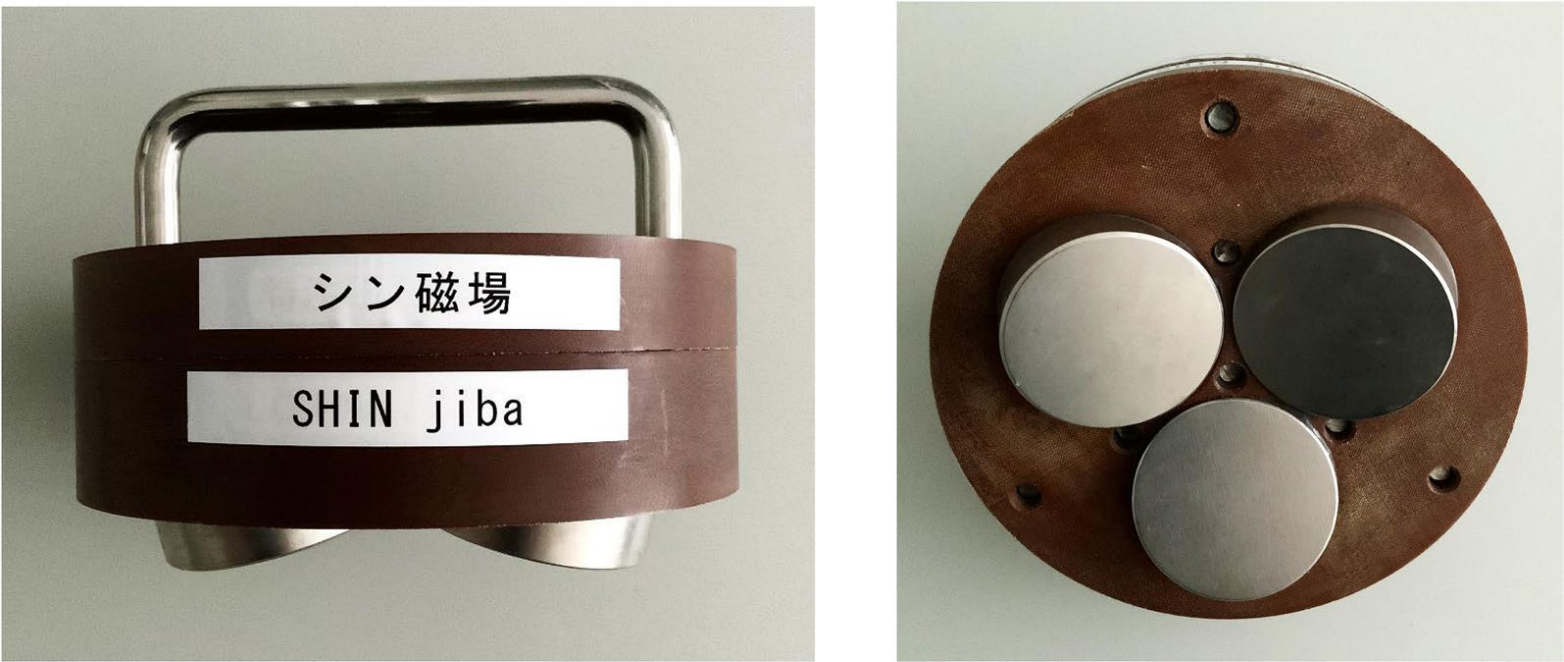
Photographs of the triple tSMS, “SHIN jiba”, system

**Fig. 2:**
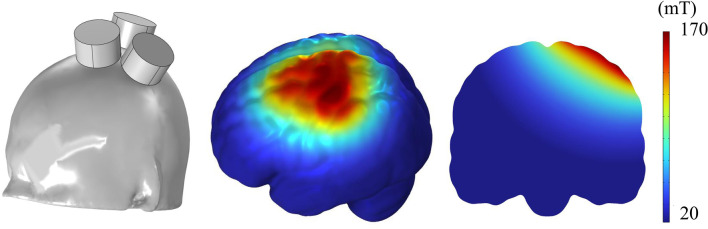
Simulated magnetic field profile in ICBM152. The triple tSMS was applied over the hand M1 on the left hemisphere. The simulation was conducted in COMSOL Multiphysics v6.0 (COMSOL, Inc., Burlington, MA, USA)

We designed and fabricated a triple tSMS system with three cylindrical nickel-plated (Ni–Cu–Ni) NdFeB magnets placed close to each other. The north pole of the three magnets were embedded in a foundation made of non-magnetic material with a diameter of 140 mm and a thickness of 48 mm. The vertical axis of the magnets was tilted 16.5 degree from that of the foundation. Parameters of the magnets were as follows: the diameter was 50 mm, the thickness was 30 mm, the maximum energy density was 406 kJ/m^3^, the nominal strength was 863 N, and the surface magnetic flux density was approximately 5340 G (Model N-50; New Mag, Sakura, Chiba, Japan) (Fig. 1). We used three magnets because of a trade-off between summation of the magnetic fields from multiple magnets and avoidance of poor focality. Figure 2 shows the spatial distribution of the magnetic field by this system generated in a human brain model (ICBM152 [14] [15]).

### Measurement of the strength of the magnetic field produced by the triple tSMS

The strength of the magnetic field produced by the triple tSMS was measured with a HTD18-0604 transverse probe and an FW Bell 5180 Gaussmeter (OECO LLC, Orlando, USA). All measurements represent the strength of the magnetic field vector along the axis perpendicular to the flat side of the probe. The decay of the static magnetic field with distance was measured by increasing the distance between the probe and the surface of the foundation, keeping the flat side of the probe parallel to the surface of the foundation. We performed duplicate measurements at the center of the foundation, that of one of the three magnets, and a point between the centers of the two magnets on the foundation. The strength was measured at intervals of 5 mm (Z-axis) from 10 mm below the surface of the foundation to a depth of 100 mm (Z = − 10 to − 100) at the center of the foundation and the point between the centers of the two magnets on the foundation, and from 15 mm below the surface of the foundation to a depth of 100 mm (Z = − 15 – − 100) at the center of one of the three magnets. For comparison with the current tSMS, we also measured the strength of the magnetic field produced by a single magnet (the current tSMS) which is same as the three magnets in the triple tSMS. The strength was measured twice at intervals of 5 mm (Z-axis) from 10 mm below the surface of the magnet to a depth of 100 mm (Z = − 10 to − 100) at the center of the magnet surface.

### Stimulation over the primary motor area with the triple tSMS

To confirm the functionality of triple tSMS, we performed a stimulation experiment in 17 healthy subjects (4 women, ages of 20–26 (mean ± standard deviation, 22.4 ± 1.3)). All subjects were right-handed as determined by the Edinburgh handedness inventory [16].

The subjects were seated in a chair during the experiment. Each subject received both real (triple tSMS) and sham stimuli. The device for the sham stimulation had the same size and appearance as the triple tSMS system except that three non-magnetic stainless-steel cylinders were embedded in the foundation. To avoid carryover effects [3], the interval between real and sham stimuli was more than three days. The stimulation performed on the first day was assigned randomly among the subjects, and they were blinded to the type of stimulation. Triple tSMS (or sham) was held using an arm-type light stand (Avenger C-stand and Super Clamp; Manfrotto, Cassola, Italy) over the representational field of the right first dorsal interosseous (FDI) muscle identified by TMS (the left M1). After the position of the left M1 was marked on the scalp, we visually confirmed that the center of the foundation was located just above the mark. The intervention duration was set to 20 min [8, 9].

The protocol of TMS was same as a previous study [9] as outlined below: single-pulse and paired-pulse TMS over the left M1 was performed using a flat figure-of-eight coil (Magstim Co., Whitland, UK). The motor evoked potential (MEP) was recorded from the right FDI muscle (Nihon-Santeku Co., Osaka, Japan). The resting motor threshold (rMT) of the right FDI muscle was determined as the minimum stimulator output required to elicit an MEP of > 50 μV peak-to-peak amplitude in at least five of 10 consecutive trials [17]. The intensity of the test stimulus was adjusted to elicit an MEP of about 1 mV from the right FDI muscle before the intervention (SI1mV). Paired-pulse stimuli were applied with a subthreshold conditioning stimulus (CS) at 80% of the rMT followed by a suprathreshold test stimulus (TS) at SI1mV with interstimulus intervals (ISIs) of 3 and 12 ms to examine short-latency intracortical inhibition (SICI) and intracortical facilitation (ICF), respectively. The paired-pulse stimuli mixed with single-pulse stimuli at SI1mV (unconditioned stimulus) were applied in a pseudo-random order. In each block, 15 trials were recorded for each of three conditions (single pulse stimuli and paired-pulse stimuli with ISIs of 3 and 12 ms). Thus, a total of 45 stimuli were applied in each block. The inter-trial interval was set at 4, 5, or 6 s in a pseudo-random order. The same test and conditioning intensities were used for both of the blocks (Baseline and Post).

After visually removing trials containing significant artifacts, we measured the peak-to-peak MEP amplitudes and calculated the averages. To evaluate corticospinal excitability in the left M1, the mean unconditioned MEP amplitude at Post was normalized to that at Baseline. For parameters of SICI and ICF, the amplitude ratio of the mean conditioned (with preceding CS) MEP to the mean unconditioned (TS alone) MEP was calculated. All the values were transformed to logarithm.

## Results

### Magnetic field profile of the triple tSMS

**Fig. 3:**
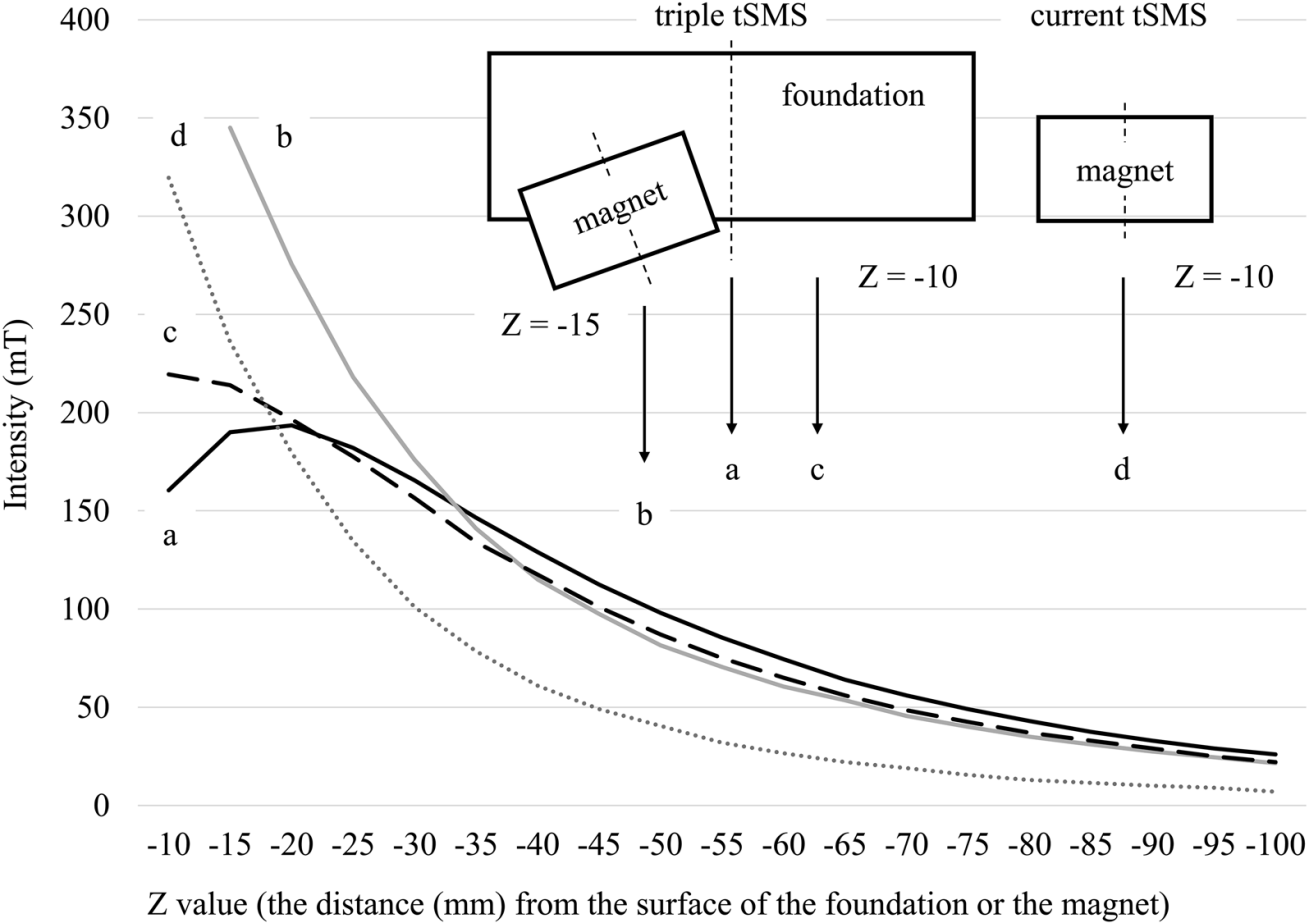
Magnetic field profile of the triple tSMS. Measured 2D magnetic field profile of the triple tSMS at the center of the foundation (**a**), the center of one of the three magnets (**b**), a point between the centers of the two magnets on the foundation (**c**), and that of the current tSMS at the center of the magnet (**d**)

For the measurements at the center of the foundation, the strength–depth curve was unimodal (Fig. 3a). There was a rapid peak (193.5 mT) at 20 mm depth, followed by a gradual decline. The magnetic field was > 40 mT up to a depth of 80 mm, which was reported to be sufficient to influence synaptic neurotransmitter release [18]. On the other hand, the magnetic field produced by the single magnet rapidly decreased along the depth and fell below 40 mT at 55 mm depth (the magnetic field was > 40 mT up to a depth of 50 mm) (Fig. 3d). This result of the single magnet is in concurrence with a previous study using an NdFeB magnet with a nominal strength of 765 N [19].

For the measurements at the center of one of the three magnets and the point between the centers of the two magnets on the foundation, the strength of the magnetic field decreased monotonically as the depth increased (Fig. 3b and c, respectively).

### Neuromodulation of human motor cortex

At the end of each experimental session, the subjects were asked what type of stimulation they received. In the real tSMS session, 13 answered that they were not sure, 4 subjects thought that they received real stimulation, and none thought that they received sham stimulation. In the sham tSMS session, 14 answered that they were not sure, 2 subjects thought that they received sham stimulation, and 1 thought that they received real stimulation.

**Fig. 4:**
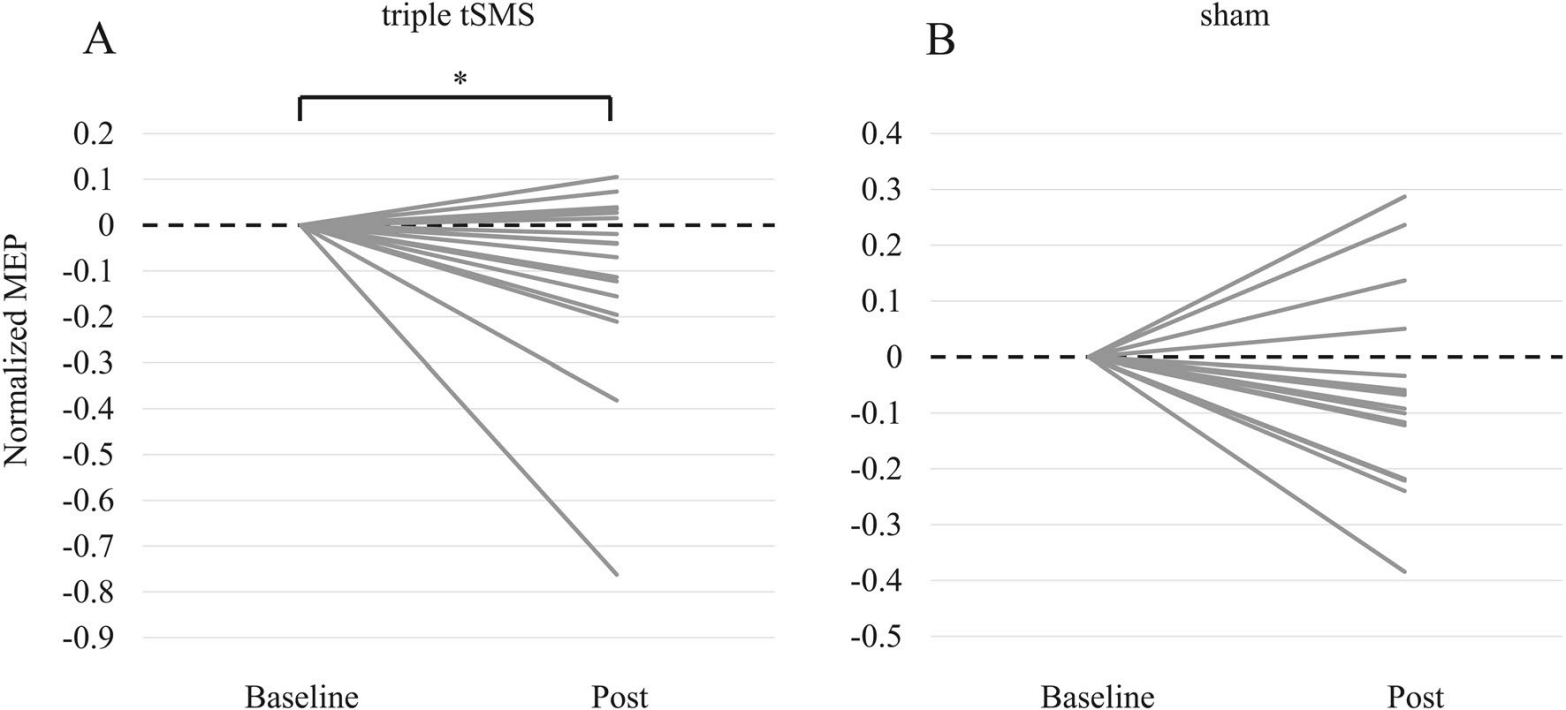
The change of the MEP elicited by single pulses of TMS. The normalized MEP significantly decreased after the intervention in the triple tSMS session (*r* = 0.50, *p* = 0.039) (**A**), but not in the sham session (*r* = 0.38, *p* = 0.113) (**B**)

In the real tSMS session 11 (64.7%) showed a decrease in the MEP after the intervention, while in the sham tSMS session 13 (76.5%) showed a decrease in the MEP. Wilcoxon signed-rank test between Baseline and Post showed that the normalized MEP in the triple tSMS session (median -0.04, interquartile range − 0.18–0.03) significantly decreased after the intervention (*r* = 0.50, *p* = 0.039), while that in the sham session (median − 0.07, interquartile range − 0.17–0.01) did not change significantly (*r* = 0.38, *p* = 0.113) (Fig. 4).

**Fig. 5:**
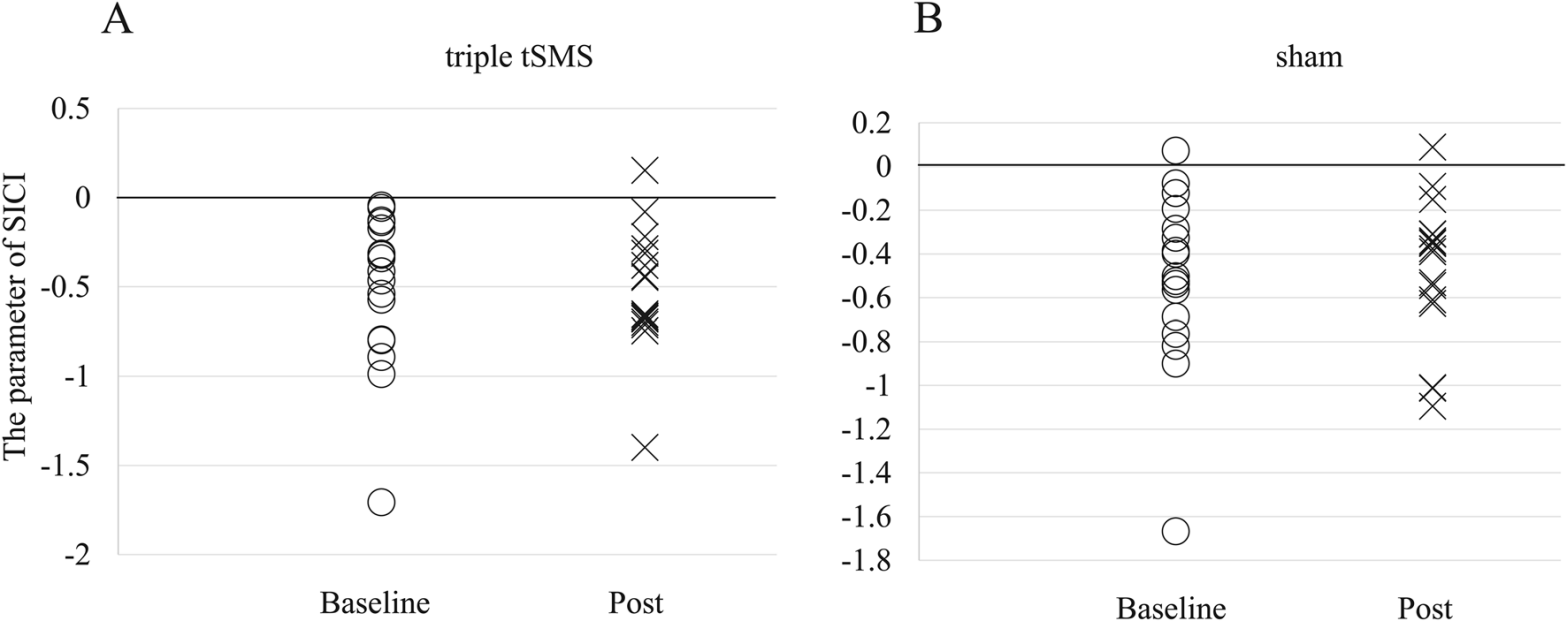
The change of the parameter of SICI between Baseline and Post. (**A**) Wilcoxon signed-rank test showed there was no difference (*r* = 0.09, *p* = 0.795) between Baseline and Post in the triple tSMS session (Baseline: median − 0.41, interquartile range − 0.80 to − 0.15, Post: median − 0.65, interquartile range − 0.69 to − 0.30). (B) Wilcoxon signed-rank test showed there was no difference (*r* = 0.05, *p* = 0.831) between Baseline and Post in the sham session (Baseline: median − 0.50, interquartile range − 0.73 to − 0.24, Post: median − 0.38, interquartile range − 0.62 to − 0.31)

**Fig. 6:**
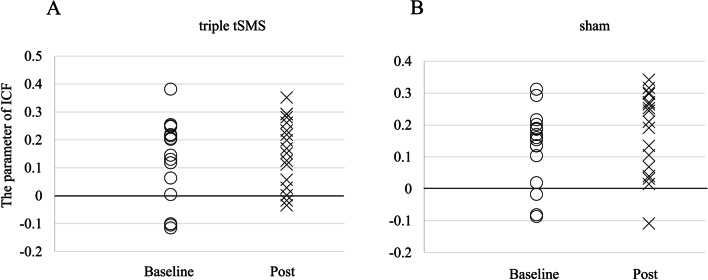
The change of the parameter of ICF between Baseline and Post. **A** Wilcoxon signed-rank test showed there was no difference (*r* = 0.10, *p* = 0.492) between Baseline and Post in the triple tSMS session (Baseline: median 0.15, interquartile range 0.03–0.23, Post: median 0.17, interquartile range 0.04–0.27). **B** Wilcoxon signed-rank test showed there was no difference (*r* = 0.37, *p* = 0.124) between Baseline and Post in the sham session (Baseline: median 0.16, interquartile range 0.06–0.20, Post: median 0.21, interquartile range 0.05–0.28)

For SICI there was no significant difference between Baseline and Post either in the real or sham session (Fig. 5). For ICF there was no significant difference between Baseline and Post either in the real or sham session (Fig. 6).

## Discussion

By devising the triple tSMS system, nicknamed “SHIN jiba”, we have demonstrated a possibility of non-invasive deep brain stimulation for human (in Japanese shin means “brand-new”, “deep”, and “authentic”; jiba means “magnetic field”). Although more efforts are required to investigate the mechanisms of the neuromodulation by the static magnetic field, the proposed system can provide not only the same neuromodulatory effect as that of the current tSMS [2] but also a new capability of enabling to produce an effective magnetic field in deep brain areas.

The measurement of the magnetic field profile showed that the triple tSMS system produced the magnetic field sufficient for neuromodulation up to 80 mm depth from the surface of the foundation. Just below the system, the magnetic field was weaker for the triple tSMS system than the current tSMS due to the interference of the magnetic fields produced by the three magnets. However, considering the distance (approximately 20 mm) between the scalp and the superficial cerebral cortex [2], the triple tSMS system placed directly on the scalp would produce at least 100 mT of the magnetic field on the superficial cortex just below the center of the system.

The stimulation experiment showed that the triple tSMS inhibited the excitability of the human motor cortex. This neuromodulatory effect is comparable to the current tSMS [2, 6, 20–22]. The neuromodulatory changes due to a static magnetic field (SMF) exposure have been investigated. A past study suggested that the SMF induces a reorientation of membrane phospholipids via diamagnetic anisotropy and alters functions of ion channels within the cell membrane [23]. A recent study also proposed another hypothesis that the magnetic field gradient produced by a SMF can induce surface tensions altering the gating probability of mechanosensitive channels [24]. Although the exact mechanism of actions mediated by the SMF remains to be elucidated, the SMF has an impact on cellular systems [25]. In fact, tSMS can decrease the cortical excitability in various areas other than the motor cortex in humans [4, 26].

In this study the triple tSMS showed different effects on the intracortical neural circuits as compared to the current tSMS. The triple tSMS induced no significant changes in SICI or ICF, while the current tSMS over the M1 for 20 min increased SICI but had no effect on ICF [6]. The possible explanation for this partial difference could be a difference in the magnetic profiles produced by these systems. Although application of the current tSMS for 10–20 min increased SICI, that for 30 min decreased SICF [22]. It was proposed that the current tSMS for short duration might act by decreasing glutamatergic excitation, while that for long duration might decrease both glutamatergic excitation and GABA (gamma-aminobutyric acid) -ergic inhibition [22]. Further studies are needed to assess how stimulation duration influences the neuromodulatory effects of the triple tSMS.

## Conclusion

Although the triple tSMS system has poor focality than the current tSMS, its neuromodulatory effect is comparable to the current tSMS. In addition, it has a capacity to produce an effective magnetic field in deep areas. Expanding the effective range with the triple tSMS can enhance the clinical utility of safe, simple, low-cost tSMS.

## Data Availability

The datasets used and/or analysed during the current study are available from the corresponding author on reasonable request.

## Abbreviations

CS: Conditioning stimulus
DBS: Deep brain stimulation
FDI: First dorsal interosseous
GABA: Gamma-aminobutyric acid
ICF: Intracortical facilitation
ISIs: Interstimulus intervals
MEP: Motor evoked potential
NdFeB: Neodymium
rMT: Resting motor threshold
SICI: Short-latency intracortical inhibition
SMF: Static magnetic field
tES: Transcranial electrical stimulation
TMS: Transcranial magnetic stimulation
tSMS: Transcranial static magnetic field stimulation
TS: Test stimulus
